# Thermal Lens Measurements of Thermal Expansivity in Thermosensitive Polymer Solutions

**DOI:** 10.3390/polym15051283

**Published:** 2023-03-03

**Authors:** Vincenzo Ruzzi, Stefano Buzzaccaro, Roberto Piazza

**Affiliations:** Department of Chemistry, Materials Science and Chemical Engineering (CMIC) “Giulio Natta”, Politecnico di Milano, Edificio 6, Piazza Leonardo da Vinci 32, 20133 Milano, Italy

**Keywords:** thermal expansivity, thermosensitive polymers, PniPAM, poloxamers, optothermal methods

## Abstract

The weak absorption of a laser beam generates in a fluid an inhomogeneous refractive index profile acting as a negative lens. This self-effect on beam propagation, known as Thermal Lensing (TL), is extensively exploited in sensitive spectroscopic techniques, and in several all-optical methods for the assessment of thermo-optical properties of simple and complex fluids. Using the Lorentz–Lorenz equation, we show that the TL signal is directly proportional to the sample thermal expansivity α, a feature allowing minute density changes to be detected with high sensitivity in a tiny sample volume, using a simple optical scheme. We took advantage of this key result to investigate the compaction of PniPAM microgels occurring around their volume phase transition temperature, and the temperature-driven formation of poloxamer micelles. For both these different kinds of structural transitions, we observed a significant peak in the solute contribution to α, indicating a decrease in the overall solution density—rather counterintuitive evidence that can nevertheless be attributed to the dehydration of the polymer chains. Finally, we compare the novel method we propose with other techniques currently used to obtain specific volume changes.

## 1. Introduction

Thermal expansivity—namely, the fractional variation of specific volume with temperature—is a powerful parameter by which to highlight and monitor structural changes and aggregation processes taking place in complex fluids. Notable examples are surfactant micellization [1,2], protein conformal transitions [3,4,5], and the thermal responses of smart materials [6,7,8,9].

Measurements of the thermal expansion coefficient in polymer systems are usually made with classic dilatometry [10], or—to detect, for instance, non-linear structural relaxations—at the glass transition, with sensitive Capacitive Scanning Dilatometry methods [11,12]. A drawback of standard dilatometric techniques, however, is that obtaining the thermal expansivity, α=−∂lnρ(T)/∂T, requires taking the numerical derivative of the sample density ρ(T), which inevitably introduces noise in data processing.

This limitation can be overcome by resorting to techniques like Temperature Modulated Differential Scanning Calorimetry (TMDSC) [13,14] or Optical Refractometry (TMOR) [15,16], which—by detecting the in-phase and quadrature components of the response to a sinusoidal temperature modulation—provide a direct measurement of α, although temperature modulation can be performed only at very low frequency, unless the sample is very small. Compared to temperature, pressure can be adjusted much faster. This is the basic strategy exploited in Pressure Perturbation Calorimetry (PPC), an ingenious technique that allows α to be directly evaluated from the heat released or absorbed when the pressure above a solution is rapidly changed [17,18,19]. Nonetheless, the need to operate with an airtight setup, typically requiring accurate pressure changes of several atmospheres, makes this approach rather elaborate.

In this paper, we show that the thermal expansivity of polymer solutions or other complex fluids can be accurately assessed by means of Thermal Lensing (TL), an all-optical technique based on the self-effect on propagation of a laser beam passing through a partially absorbing medium, which provides a fast and non-invasive tool for the direct determination of the thermal expansion coefficient in fluids. A unique feature of the TL method is that it requires a very simple basic optical and detection scheme, requiring no more than a low-power near-infrared laser, a beam shutter, a focusing lens, a thermostatted cell, and a fast photodiode.

The setup we developed and used was specifically designed to deal with aqueous solutions and suspensions—definitely the most interesting classes of systems where structural changes notably affect thermal expansivity [20]. As significant illustrative examples, we tested and validated the method on aqueous suspensions of poly-N-isopyilacrylamyde (PniPAM) microgel particles, and on solutions of two block copolymers (poloxamers) displaying a very different phase diagram: namely, Pluronic^®^ F127 and Pluronic^®^ 17R4. By increasing the temperature, the systems we investigated underwent substantial transformations: namely, a sudden compaction of the PniPAM microgels and association into micelles of the poloxamer chains. As we shall see, an attentive investigation of the temperature dependence of the thermal expansivity coefficient highlighted that these apparently unrelated effects shared a common origin in the dehydration of the polymer chains, which affected the density of the chain solvation layer. Finally, we compare the TL method to other techniques used for thermal expansivity measurements, and suggest a further application of the technique to the investigation of soft disordered solids, such as tenuous hydrogels.

## 2. Materials and Methods

### 2.1. Thermal Lens Principle and Thermal Expansivity Measurements

When a laser heats up a sample locally, thermal expansion induces a radial density distribution, which generates an inhomogeneous refractive index profile acting as a negative lens: this results in an increased divergence of the transmitted beam, which can be precisely evaluated by detecting the change in the beam-center intensity I(t) [21,22]. Consider a Gaussian laser beam of wavelength λ of optical power *P*, which is focused by a lens on a sample of thickness *l*, absorption coefficient *b*, mass density ρ, specific heat $c_{p}$, thermal conductivity κ, and thermal diffusivity $\chi =\kappa /(\rho c_{p})$. Assuming a 2D geometry that neglects heat flow along the optical axis, the heat equation leads, in the small-angle (paraxial) approximation, to a parabolic temperature profile, ΔT(r,t), which turns into a local refractive index radial distribution acting as a thin diverging lens. Including also aberrations not taken into account in the paraxial approximation, and introducing the dimensionless thermal lens number

(1) $$\vartheta_{th}=-\frac{Pbl}{\kappa \lambda }\frac{\partial n}{\partial T},$$

the time dependence of the beam-center intensity I(t) can be written as

(2) $$I\left(t\right)=\frac{I(0)}{1+\vartheta_{th}f\left(t\right)},$$

where

(3) $$f\left(t\right)=\frac{\tilde{z}}{3+{\tilde{z}}^{2}}\frac{\pi }{\sqrt{3}\left(1+\tau_{th}/t\right)}.$$

In Equation (3), $\tau_{th}=\omega^{2}/4\chi$ is the characteristic heat diffusion time over the laser beam spot size ω, and $\tilde{z}=z/z_{R}$ is the (thin) sample distance from the beam waist (the plane where the beam attains its minimal size $w_{0}$) scaled to the Rayleigh length $z_{R}=\pi w_{0}^{2}/\lambda$. Equation (2) is also a very good approximation to an exact 2D Fresnel diffraction analysis [23]. For $t\gg \tau_{th}$, f(t) attains the asymptotic value

$$f\left(\infty \right)=\frac{\pi \tilde{z}}{\sqrt{3}\left(3+{\tilde{z}}^{2}\right)},$$

which takes on its maximum value, f(∞)=π/6, when the sample is placed at a distance, $z=\pm \sqrt{3}z_{R}$, from the beam waist. In this configuration, the beam spot size is $w=2w_{0}$. With these settings, yielding a minimum value $I_{\infty }$ for the center-beam transmitted intensity, the thermal lens number is simply given by

(4) $$\vartheta_{th}=\frac{6}{\pi }\frac{I_{0}-I_{\infty }}{I_{\infty }}$$

It is worth noticing that, while in simple fluids the TL effect is only due to thermal expansion, in multicomponent systems, the laser-induced thermal gradient also causes thermodiffusion effects, generating a concentration profile in the heated region, which acts as a further lens-like element. Indeed, TL can be profitably exploited for the study of the Soret effect in fluid mixtures [24], or of thermophoresis in colloids [25]. In addition to its applications to the study of thermal diffusive phenomena, the TL technique is mainly used as a spectroscopic method in analytic chemistry, as it is able to detect extinction coefficients in simple fluids as low as $10^{-7}$ [26,27]. Furthermore, due to its high sensitivity and spatial resolution, TL finds applications in microfluidics and microscopy [28,29], and can be employed for noninvasive measurements of thermal conductivity in nanofluids [30].

It is not difficult to provide a link between the TL signal and the sample thermal expansivity α, if we recall that the refractive index is related to the fluid density, ρ, by the Lorenz–Lorentz (LL) equation,

(5) $$\frac{n^{2}-1}{n^{2}+2}=r\rho ,$$

where *r* is the optical polarizability per unit mass (also known as specific refractivity), which can be shown to be valid up to second order in (ξ/λ) for polymer solutions (or, more generally, correlated fluids) with a correlation length ξ [31]. Assuming that the specific refractivity does not appreciably vary with temperature, and taking the temperature derivative of (5), we have

(6) $$\alpha =-\frac{1}{\rho }\frac{\partial \rho }{\partial T}=-\frac{6n}{\left(n^{2}-1\right)\left(n^{2}+2\right)}\frac{\partial n}{\partial T}=\frac{6n\kappa \lambda }{Pbl\left(n^{2}-1\right)\left(n^{2}+2\right)}\vartheta_{th}.$$

In a particle suspension or polymer solution, we can gauge the contribution per unit volume of the solute to the thermal expansivity, $\alpha_{p}$, by writing

(7) $$\alpha =\alpha_{p}c{\bar{v}}_{p}+\alpha_{s}\left(1-c{\bar{v}}_{p}\right)$$

where ${\bar{v}}_{p}$ is the solute specific volume, *c* is the solute concentration in mass/volume, and $\alpha_{s}$ is the thermal expansivity of the pure solvent. We point out that the parameter $\alpha_{p}$, so defined, does not account for an inherent property of the solute, but rather summarizes its overall effect on the thermal expansivity of the solution/suspension. The physical mechanism giving rise to this effect can be, for instance, the development of a hydration layer around the solute, with structural properties different from those of the bulk solvent. Moreover, while the solute specific volume can, in principle, depend on *c*, we confirmed that, for the dilute solutions we examined, ${\bar{v}}_{p}$ did not differ by more than a few percent from the reciprocal of the bare polymer density (which, for all the systems we examined, did not differ by more that 10% from the solvent density).

**Figure 1:**
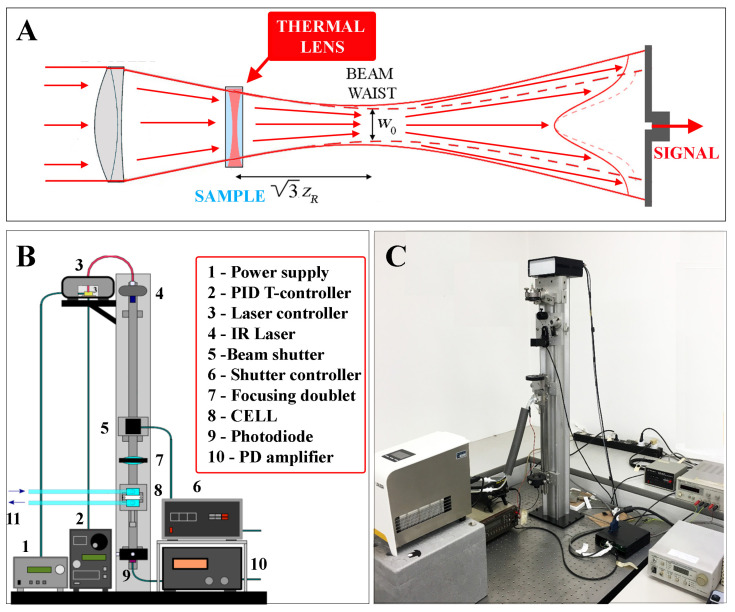
Panel (**A**) is a sketch of the TL effect, with the sample acting as a thin diverging lens that increases the divergence of the focused Gaussian beam (compare full and broken lines); the Gaussian beam expansion entails a decrease of the central beam intensity, which is detected by a photodiode placed behind a pinhole. Panels (**B**,**C**) are a schematic diagram and an actual picture of our setup, which is vertically mounted to minimize convection effects when the apparatus is used to study slow thermophoretic effects (for an extensive discussion, see [25]).

**Figure 2:**
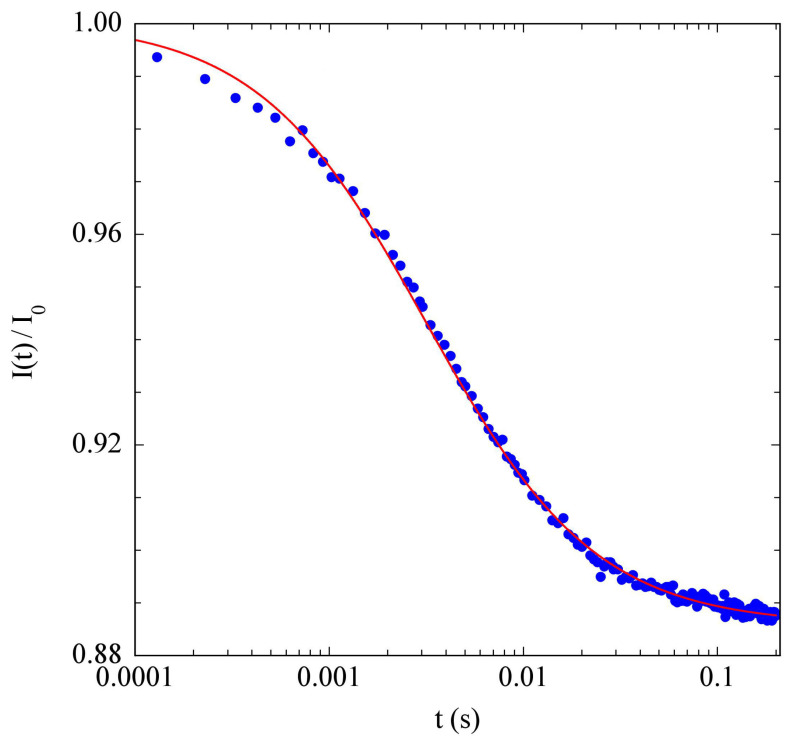
Normalized time-dependent central-beam intensity for a 1% suspension of PniPAM microgel particles at T=28 °C. The red line is a fit with Equation (2), using $\vartheta_{th}≃0.23$ and $\tau_{th}≃3\;\mathrm{ms}$).

Our custom-made TL setup, sketched in Figure 1, exploited the weak absorption by aqueous solvents of a near-infrared laser source emitting at λ=976nm, which corresponded to a vibrational overtone of water with absorption coefficient $b≃0.55\;{\mathrm{cm}}^{-1}$. The sample cell was a cylindrical cuvette with a 9mm internal diameter and an optical path l=1mm (Hellma 165QS), enclosed in an external quartz jacket allowing water to circulate from a thermostatic bath (Lauda Loop L100), and placed on a mounting that allowed micrometric translation along the optical axis, to maximize the TL signal. Detection of the center-beam intensity was by means of a fast-pinhole photodiode. Unless otherwise stated, all measurements were performed with a laser power P=40mW. As the temperature increase on the beam axis could be estimated as ΔT≃0.3Pb/κ [25], for this value of *P* we had ΔT≃1 °C. More detailed information on the TL setup can be found in [32]. A typical TL signal from a suspension of PniPAM microgel particles is shown in Figure 2.

Refractive index measurements of the analyzed aqueous solutions were made with an Abbemat RXA Digital Refractometer (Anton Paar), with a sensitivity $\Delta n=10^{-5}$.

### 2.2. Samples

PniPAM microgels, extensively used as model soft colloidal particles, are used as smart sensors in analytical chemistry [33], and as drug carriers in nanomedicine [34,35]. Here, we simply recall that PniPAM free chains have a ϑ-temperature of T≃31 °C and undergo a coil-to-globule transition at T≃32 °C, in which the collapsed structures avoid the contact between PniPAM hydrophobic parts and water. When $T<T_{\vartheta }$, the microgel particles swell in water, and the chains are well-hydrated; for $T>T_{\vartheta }$, temperature effects on the single chains cause a sharp contraction of the microgel particles, with a reduction of the hydrodynamic radius $R_{H}$, characterized by a shrinkage rate that is maximum at the so-called volume phase transition temperature (VPTT). The molecular mechanisms behind the VPTT were unraveled by super-resolution microscopy [36] and UV resonant Raman [37], and substantiated by molecular simulations [38,39]. The PniPAM microgel particles we used were a kind gift by Kodak European Research, and were previously used to investigate thermophoresis by Wongsuwarn et al. [40], to whom we refer for an extensive discussion of their physical properties. Here, it is only useful to recall that the hydrodynamic radius of these microgels decreases from about 150nm at T=20 °C to 60nm at T=40 °C, decreasing then by more than 15 times in volume across the VPTT.

Poloxamers, commercially known as Pluronics^®^, are low-toxic, biocompatible block copolymers constituted by alternating hydrophilic–hydrophobic blocks of PolyPropyleneOxide (PPO) and PolyEthyleneOxide (PEO), thus showing temperature-dependent amphiphilic behavior caused by the increasing hydrophobicity of the PPO block with temperature and a rather complex phase behavior. In particular, Pluronics^®^ display a concentration-dependent critical micellar temperature (CMT), over which the unimers self-assemble into micelles, embedding the PPO and exposing the PEO group to water. Pluronic^®^ F127 has a chemical structure with a central PPO block and the two PEO hydrophylic side chains. Micellization properties and phase behavior have been deeply investigated by means of Dynamic Light Scattering (DLS), Small-Angle Neutron Scattering (SANS), rheology [41,42,43], and also TL [32]. Pluronic^®^ 17R4 is conversely made of an internal hydrophilic PEO block joining two hydrophobic side PPO chains. By increasing the temperature, the 17R4 solution becomes progressively blueish and turbid, presumably because of pre-aggregation processes taking place before micellization, which lead to the formation of clusters coexisting with the unimers [44]. Above the CMT, however, these clusters disappear, and “flower” micelles form, where the PPO blocks fold in the micellar core, leaving the PEO part exposed to water (a micellization mechanism typical of the so-called “reverse Pluronics” [45,46]). Hence, the solution turned back to full transparency up to about 40–45 °C, where phase separation eventually took place [47,48]. The poloxamers we investigated were purchased from Sigma–Aldrich, and were used without further purification.

## 3. Results

### 3.1. PniPAM Microgels

**Figure 3:**
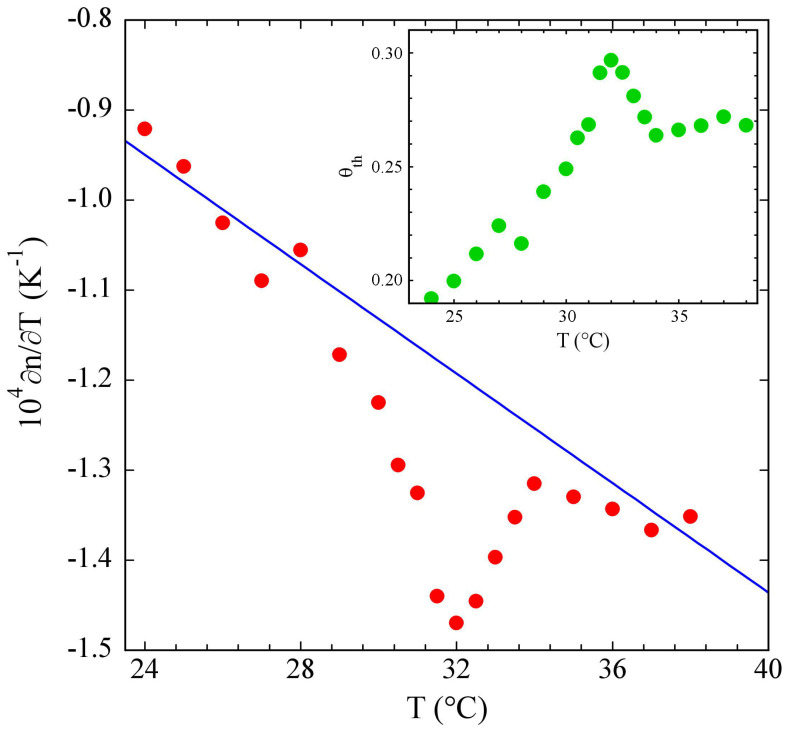
Temperature dependence of ∂n/∂T for a suspension of PniPAM microgel particles at 1% concentration, showing a negative peak around T=32 °C. The line shows the linear decrease of ∂n/∂T for pure water in the same temperature range. Values of the thermal lens number for PniPAM are shown in the inset.

The temperature derivative of the refractive index of a solution of PniPAM microgels at a particle concentration of 1%, obtained from the thermal lens number $\vartheta_{th}\left(T\right)$ displayed in the inset, is shown in Figure 3. While at lower and higher temperatures its values were reasonably close to those for pure water (full line), in the temperature region around the VPPT a consistent negative peak showed up, leading to a decrease of about 25% in ∂n/∂T at T=32 °C.

**Figure 4:**
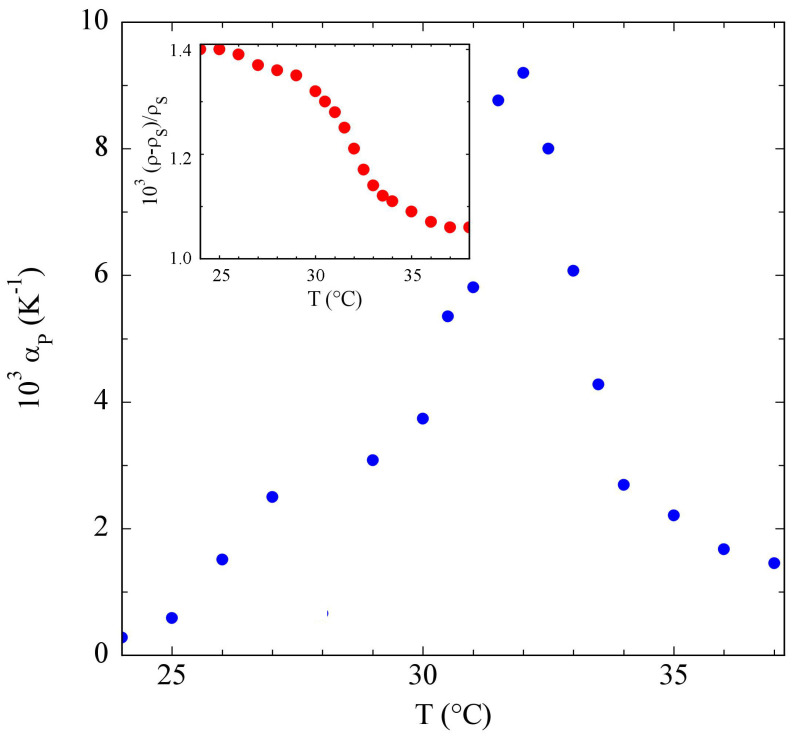
Particle contribution $\alpha_{p}$ to the thermal expansivity for a 1% PniPAM solution, showing a pronounced peak around the VPTT. Inset: temperature dependence of the excess density, $\left(\rho -\rho_{s}\right)/\rho_{s}$, calculated according to Equation (8), using ${\left(\rho /\rho_{s}\right)}_{T=24\;{}^{\circ}C}≃1.00014$.

Using Equation (7), with ${\bar{v}}_{p}≃0.91\;{\mathrm{cm}}^{3}/g$ for the PniPAM specific volume, we extracted the specific contribution $\alpha_{p}$ of the PniPAM microgel particles to the thermal expansivity. The body of Figure 4 highlights that $\alpha_{p}$ had a sharp maximum at the VPTT, in agreement with other evidence of the thermoresponsivity of PnIPAAM aqueous solutions, both as free chains [16,17] and as crosslinked colloidal particles [49,50]. It is also easy to show that the ratio $\rho /\rho_{s}$ between the solution and the solvent density could be simply obtained by a numerical integration of $\Delta \alpha =\alpha -\alpha_{s}$,

(8) $$\frac{\rho }{\rho_{s}}={\left(\frac{\rho }{\rho_{s}}\right)}_{T=T_{0}}\exp\left(-\int_{T_{0}}^{T}\Delta \alpha \left(T^{\prime }\right)dT^{\prime }\right).$$

The inset of Figure 4 shows that $\rho /\rho_{s}-1$ displayed a small but sharp decrease around the VPTT, saturating for T≳38 °C to a value that was about $4\times 10^{-4}$ lower than at low temperature.

It may be surprising that the microgel compaction due to the volume phase transition led to a decrease of the solution density. The origin of this peculiar effect can, however, be grasped if we realize that microgel collapse was associated with a consistent dehydration of the PniPAM chains, which, at the VPTT, partly lost the highly coordinated solvent shell that surrounded them at low temperatures [38]: hence, the increase of the solvent specific volume and the peak in the thermal expansivity were arguably due to the involved entropy increase of the water molecules.

**Figure 5:**
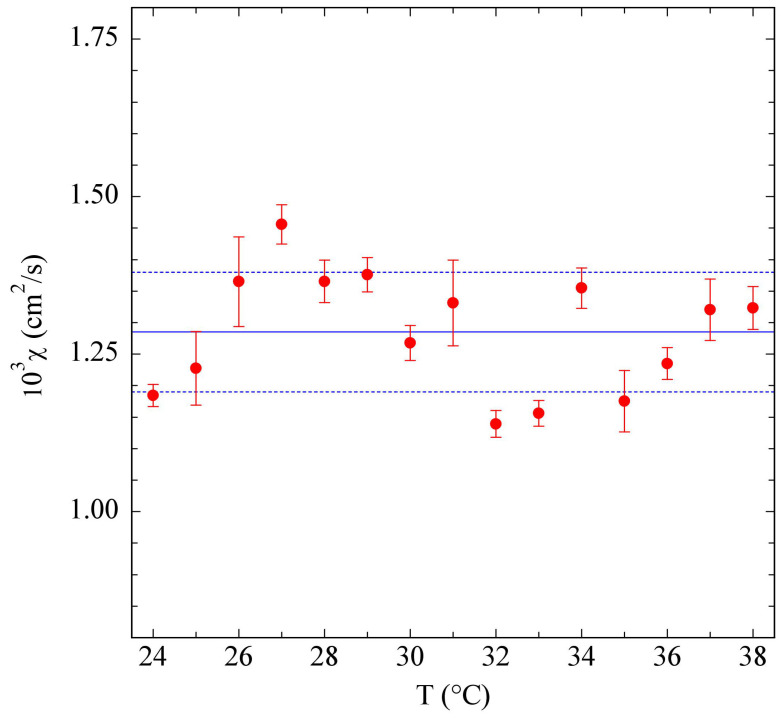
Temperature dependence of the thermal diffusion coefficient χ of a 1% PniPAM solution. Full and dashed lines show the average value plus/minus one standard deviation.

Finally, it is useful to investigate the dynamics of the thermal lens, a fit of which, as shown by Equations (2) and (3), directly yielded the sample thermal diffusivity χ. Figure 5 shows that the structural effects highlighted by the peak in the thermal expansivity did not reflect in any relevant change of a transport property like χ, which was to a good approximation constant in the investigated temperature range, with a value $\chi =1.29\pm 0.10\times 10^{-3}\;{\mathrm{cm}}^{2}/s$.

### 3.2. Poloxamers

**Figure 6:**
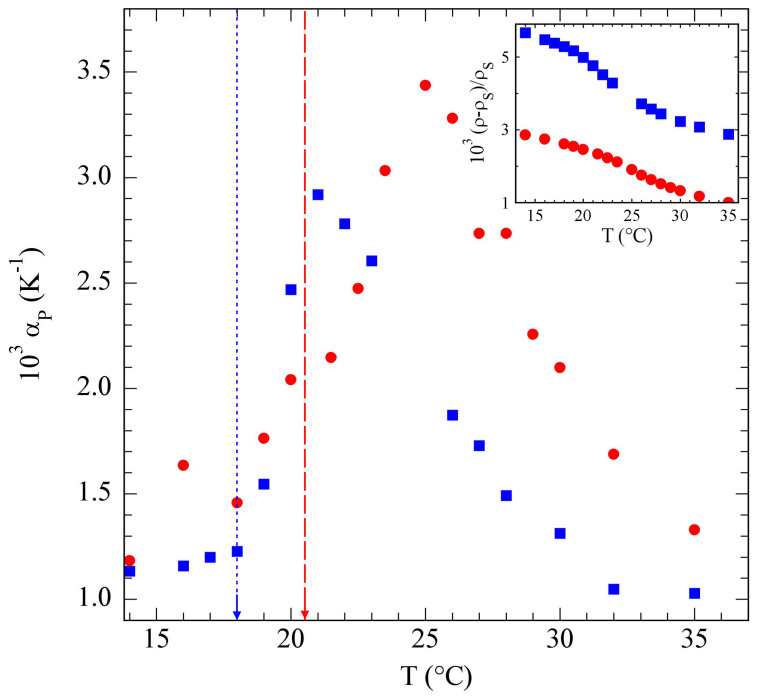
Particle contribution $\alpha_{p}$ to the thermal expansivity for c=5% (bullets) and c=10% (squares) Pluronic^®^ F127 aqueous solutions, using Equation (7) with ${\bar{v}}_{p}≃0.94$. The CMTs obtained by DLS in [32] are shown by a dashed and a dotted line, respectively. Inset: temperature dependence of the excess density, $\left(\rho -\rho_{s}\right)/\rho_{s}$, for the same solutions.

The dehydration effect we pointed out for PniPAM around the VPTT is supposed to occur in other systems, like poloxamers, which show micellization processes that strongly depend on temperature. The TL results presented in Figure 6 for Pluronic^®^ F127 [32] actually show a distinctive peak in the thermal expansivity, which started to rise at about the poloxamer CMT, and decreased with poloxamer concentration. As in PniPAM at the VPTT, the formation of Pluronic^®^ micelles was indeed driven by the partial dehydration of the PO chains with increasing temperature [51].

**Figure 7:**
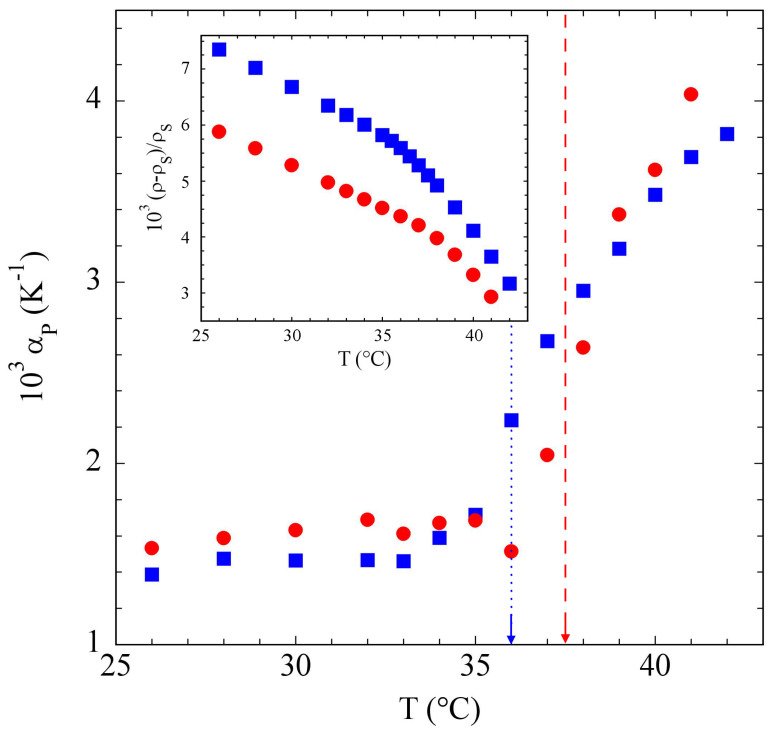
Particle contribution $\alpha_{p}$ to the thermal expansivity for c=12% (bullets) and c=15% (squares) Pluronic^®^ 17R4 aqueous solutions, using Equation (7), with ${\bar{v}}_{p}≃0.95$. The CMTs obtained in [44] are shown by a dashed and a dotted line, respectively. Inset: temperature dependence of the excess density, $\left(\rho -\rho_{s}\right)/\rho_{s}$ for the same solutions.

The results for Pluronic^®^ 17R4 were somewhat different. Figure 7 shows that, for both the concentrations we studied, $\alpha_{p}$ did start to rise around the CMT, but then steadily grew up to the maximum temperature we managed to investigate before the sample turbidity associated with the onset of phase separation became too large to detect a significant TL effect on the transmitted beam. Consequently, the solution density excess, shown in the inset, monotonically decreased over the whole temperature range, showing no sign of saturation to a lower plateau value. Such different behavior was arguably related to the different association process of this poloxamer with a “reversed” structure, which progressively occurred over a broad temperature range extending up to the cloud point where liquid–liquid phase separation took place [52]. SANS measurements moreover confirmed that the 17R4 association into “flower” micelles was driven by a progressive dehydration process taking place over the temperature range 35 °C<T<42 °C [44].

## 4. Discussion

As discussed in the introductory section, several experimental methods to obtain the thermal expansivity of complex fluids already exist: the most sensitive among them are often based on introducing a temperature modulation in a densitometer, calorimeter, or optical refractometer, and detecting the in-phase and quadrature components of the signal, a strategy that has long been exploited in electronic lock-in amplifiers. Measurements of this kind allow the sample thermal expansivity to be directly obtained, with no need to take a numerical derivative of a data set, as in more traditional standard techniques. However, even for miniature samples, the largest *T*-modulation frequency that can be set by thermoelectric modules does not usually exceed a few tenths of a hertz, which makes phase-sensitive detection very time-consuming. In addition, a nontrivial trade-off between sensitivity, which increases with the modulation depth, and resolution in temperature, which conversely is reduced by increasing ΔT(t), is crucial.

When compared with these techniques, the TL method presents a key advantage. Indeed, as shown by Equation (6), the TL signal obtained by generating in a sample a weak and spatially localized step increase of the temperature is directly proportional to α. In addition, as the TL signal reaches a steady state on the timescale of heat diffusion time $\tau_{th}$ over the beam spot size (a few hundreds of milliseconds), TL measurements can be performed in a very short time on a tiny sample volume.

Currently, our TL setup detects changes in ∂n/∂T with respect to water of the order of $10^{-6}\;K^{-1}$ that, for a solute with $\partial n/\partial T≃10^{-4}\;K^{-1}$, allow us to measure samples with *c* not much lower than a few percent. The setup sensitivity can be enhanced using a dual-beam TL setup exploiting a pump-and-probe scheme, which uses a probing laser beam in the visible range. It is also useful to point out that, in this configuration, TL may easily lend itself to temperature modulation, as this simply amounts to modulating the intensity of the pump laser beam, which can be done very rapidly, thus further increasing the sensitivity of the technique.

Although the investigation we have presented is mostly meant to be illustrative of the TL method for obtaining thermal expansivity, it is, nevertheless, interesting to point out that the evidence we found shows that effects in principle as different as PniPAM compaction at the VPTT and poloxamer micellization, share a common physical origin. Further improvement of the technique sensitivity along the lines sketched above may allow us to investigate much subtler effects of primary biological interest, such as protein unfolding. As extensively discussed in [5,53], differences in the thermal expansivity between the folded and the unfolded state may originate from several causes, but a “hydrophobic collapse” similar to the one we have discussed surely plays a key role. The TL technique can be also be used to study the gelation of polymer solutions driven by temperature-dependent attractive interchain interactions: to this end, we are currently investigating the change of thermal expansivity upon gelation of Mebiol^®^, a thermoresponsive block copolymer extensively used as a substrate for cell growth [54,55]. Very interestingly, preliminary results show that in the gel phase a strong IR irradiation may actually induce permanent structural deformations, indicating the transition from elastic to plastic response of the gel.

## Data Availability

The data underlying the results presented in this paper are not publicly available at this time, but may be obtained from the authors upon reasonable request.

## Abbreviations

The following abbreviations are used in this manuscript:

TL	Thermal Lensing
PniPAM	poly-N-isopyilacrylamyde
VPTT	Volume Phase Transition Temperature
PPO	PolyPropyleneOxide
PEO	PolyEthyleneOxide
CMT	Critical Micellization Temperature
DLS	Dynamic Light Scattering
SANS	Small-Angle Neutron Scattering
